# Posterior Approach Resection of Giant Schwannomas at the Craniovertebral Junction: A Case Series

**DOI:** 10.7759/cureus.92338

**Published:** 2025-09-15

**Authors:** Glennie Ntsambi, Israël A Maoneo, Punga Ziko, Jean-Richard Kabongo, Simon Kutoloka, Chérubin Tshiunza, Larrey Kasereka Kamabu, Bienvenu Lebwaze, Raphael Chirimwami, Dieudonné Sanduku, Antoine Beltchika

**Affiliations:** 1 Neurosurgery, University of Kinshasa, Kinshasa, COD; 2 Neurosurgery, University of Kisangani, Kisangani, COD; 3 Neurosurgery, Centre Hospitalier Initiative Plus, Kinshasa, Kinshasa, COD; 4 Neurosurgery, Catholic Unversity of Graben, Butembo, COD; 5 Neurosurgery, New Deal Hospitals/CIMAK (Clinique Internationale de Médecine Avancée au Kivu), Goma, COD; 6 Neurosurgery, Makerere University, Kampala, UGA; 7 Anatomical Pathology, University of Kinshasa, Kinshasa, COD; 8 Surgical Gastroenterology, University of Kinshasa, Kinshasa, COD

**Keywords:** cranio-cervical junction tumor, extradural tumor, schwannoma, slow spinal cord compression, spinal surgery, spinal tumors

## Abstract

Tumors of the craniovertebral junction (CVJ) are rare and present a major surgical challenge due to the anatomical complexity of the region. We report three cases that were admitted and operated on in our department. All three patients presented with motor deficits and bladder-sphincter dysfunction. In the first case, MRI revealed a grossly ovoid tumor measuring 30 x 28 mm, located anterolaterally to the right of the cervical spinal cord at the level of the C2 vertebral body, extending anteriorly beyond the arch of C1. The second patient’s contrast-enhanced MRI showed an intradural extramedullary lesion measuring 31 x 27 mm at the C2-C3 level. In the third case, imaging demonstrated spinal cord compression at the C1-C2 level due to a mass measuring 35 x 26 mm in the extradural and extramedullary space, with spontaneous signal located anterior to the dural sac. All patients underwent posterior decompression. Tumor excision was performed under a neurosurgical microscope following C2 laminectomy and partial resection of the posterior arch of C1 in all three cases. Histopathological examination of the resected specimens confirmed the diagnosis of schwannomas. Postoperative outcomes were uneventful, and all patients showed improvement following physiotherapy sessions. This case series highlights the surgical complexity of the posterior approach to CVJ tumors, particularly giant schwannomas, and demonstrates the favorable postoperative course following complete resection.

## Introduction

The craniovertebral junction (CVJ), the anatomical union of the clivus, foramen magnum, and the C1 and C2 vertebrae, represents a structurally complex region. Tumors arising in this area pose a significant surgical challenge due to the dense concentration of neurovascular structures and the narrow operative corridor, which limits optimal tumor resection [1-3]. The surgeon must account for three essential principles: preserving spinal stability, maintaining mobility, and avoiding injury to critical neurovascular structures, such as the vertebral arteries, the cervicomedullary junction, and the first two cervical nerve roots, which are particularly sensitive to shear forces [4]. A favorable outcome requires a thorough clinical assessment, accurate imaging interpretation, detailed knowledge of regional anatomy, and histological diagnosis [3].

Schwannomas are heterogeneous tumors originating from Schwann cells responsible for myelination of cranial and peripheral nerves. According to the 2021 WHO classification, schwannomas are distinguished from malignant peripheral nerve sheath tumors (MPNSTs) and melanotic schwannomas due to their benign nature and distinct genetic features [5].

Craniocervical junction schwannomas are rare entities that may involve the jugular foramen, hypoglossal canal, or the C1-C2 foramina, with direct extension into the atlanto-occipital and atlanto-axial joints. They account for approximately 8% of nerve sheath tumors, although their incidence varies based on location and nerve involvement, most commonly affecting the facial and vestibular nerves [2]. Giant schwannomas are defined as those extending vertically over more than two vertebral bodies [6] or measuring more than 2.5 cm in diameter [7]. Clinical and radiological presentations are highly variable. Most of these tumors result in debilitating neuropathies and neurological deficits, frequently presenting with a characteristic dumbbell-shaped morphology [8,9].

Schwannomas of the CVJ are often misdiagnosed due to their rarity, indolent growth, and occurrence within a spacious and anatomically complex region. They are frequently asymptomatic, and when symptomatic, their signs are nonspecific, often mimicking other common lesions such as meningiomas, vestibular schwannomas, spinal nerve neurinomas, neurofibromas, malignant peripheral nerve sheath tumors, cartilage tumors (chondromas, chondrosarcomas), glomus tumors (paragangliomas), and bone or meningeal metastases [10].

Multiple surgical approaches have been described to enhance safety and facilitate careful dissection of tumor tissue from the involved nerves, particularly through precise exposure and control of vital neurovascular structures [11,12]. Common surgical corridors for CVJ tumors include posterior, transcondylar, and transoral approaches.

Giant craniovertebral schwannomas present unique surgical challenges due to their large size, invasive growth patterns with bone erosion and soft tissue involvement, and location within a highly complex anatomical region densely packed with critical cranial nerves and vascular structures. These factors often necessitate complex surgical approaches and reconstructions to achieve complete tumor removal while maintaining spinal stability and preserving neurological function. However, the risk of significant perioperative complications remains high [13].

The evolution of spinal instrumentation has provided not only a safer treatment option but also long-term surgical stability while patients undergo adjuvant therapies [3]. However, in resource-limited settings, posterior approaches to anterior or anterolateral tumors remain a high-risk strategy. Available data from sub-Saharan Africa are scarce. Here, we report three cases to discuss the socio-demographic, clinical, imaging, and therapeutic aspects of giant schwannomas of the craniocervical junction.

## Case presentation

Case 1

A 46-year-old male patient (Patient 1) was brought by his family with a history of quadriparesis, constipation, and urinary incontinence. The symptoms began approximately three months prior to admission, initially presenting as a sensation of heaviness in both hands. He sought care at a peripheral hospital where he received neurotropic vitamins without clinical improvement. Over time, he developed progressive weakness affecting all four limbs, more pronounced in the lower limbs, prompting referral to the University Teaching Hospital of Kinshasa for specialized care. His past medical history was unremarkable.

On admission, the patient was alert, with stable vital signs. He had pink palpebral conjunctivae and anicteric sclerae. Pupils were equal, reactive, and isochoric. The general condition was compromised by a bedridden state and apparent weight loss. Cervical examination revealed limited neck flexion up to 30° and pain on neck movement.

**Figure 1:**
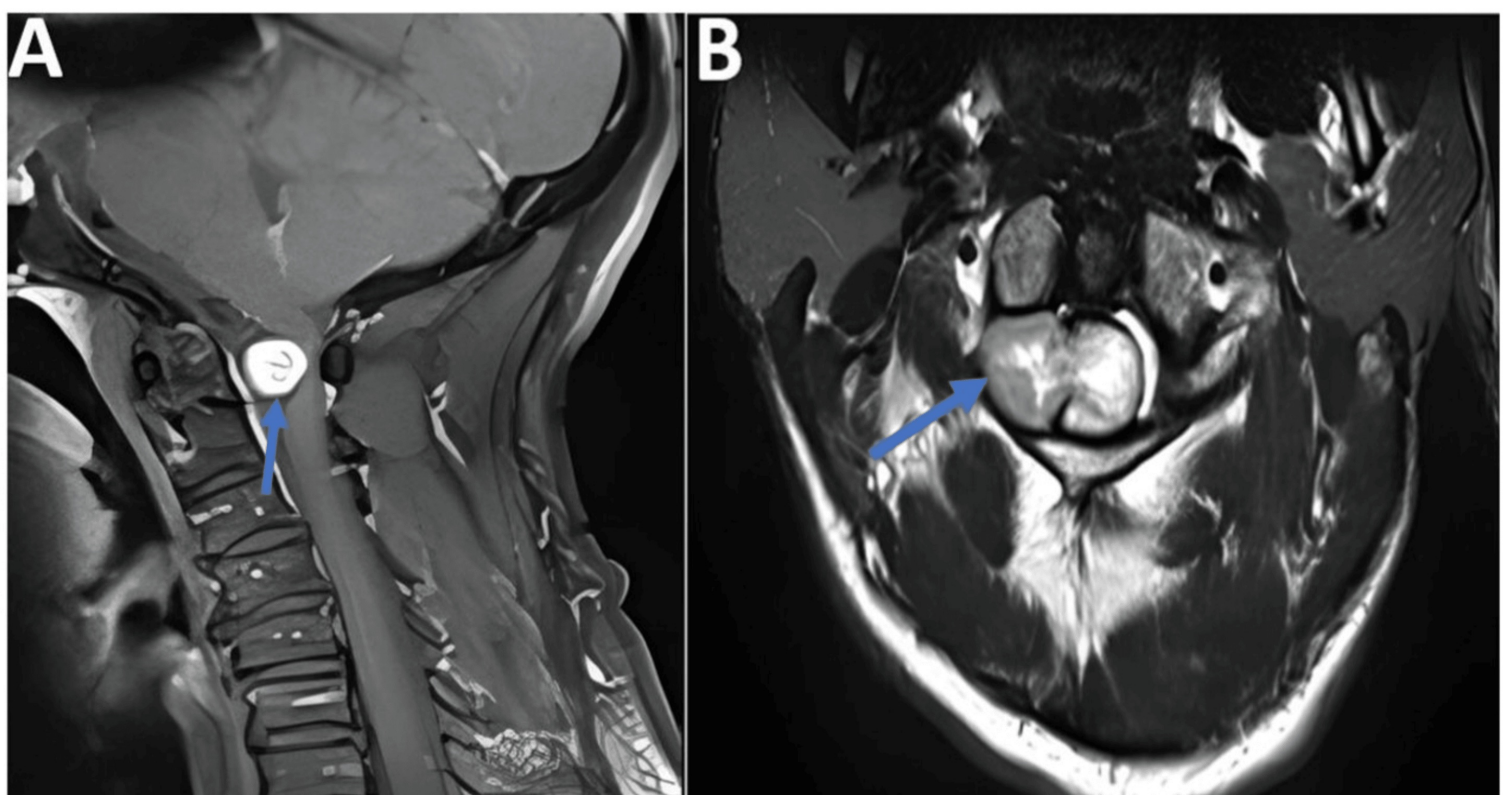
(A) Sagittal T1-weighted MRI showing an anterior intradural extramedullary mass at the level of C2 and C1; (B) Axial T1-weighted MRI showing a right anterolateral intradural extramedullary mass in contact with the C2 nerve root (blue arrow).

Neurological examination of the upper limbs showed muscle atrophy with motor strength graded at 4/5 on the right and 3/5 on the left. Sensation was preserved. In the lower limbs, there was notable atrophy with severe paraparesis graded at 3/5, accompanied by hypoesthesia. A urinary catheter was in place, draining clear yellow urine. Cervical spine MRI demonstrated a right anterolateral, ovoid tumor at the craniocervical junction, compressing the spinal cord. The lesion measured approximately 30 mm in transverse and 28 mm in vertical diameter, located at the level of C2 and extending beyond the anterior arch of C1 (Figure 1).

Case 2

A 63-year-old female patient (Patient 2) presented to the Neurosurgery Department with a six-month history of cervical pain, upper limb weakness, and paresthesia. Neurological examination revealed left upper limb monoplegia, with muscle strength graded at 3/5. No visible muscle atrophy was noted.

**Figure 2:**
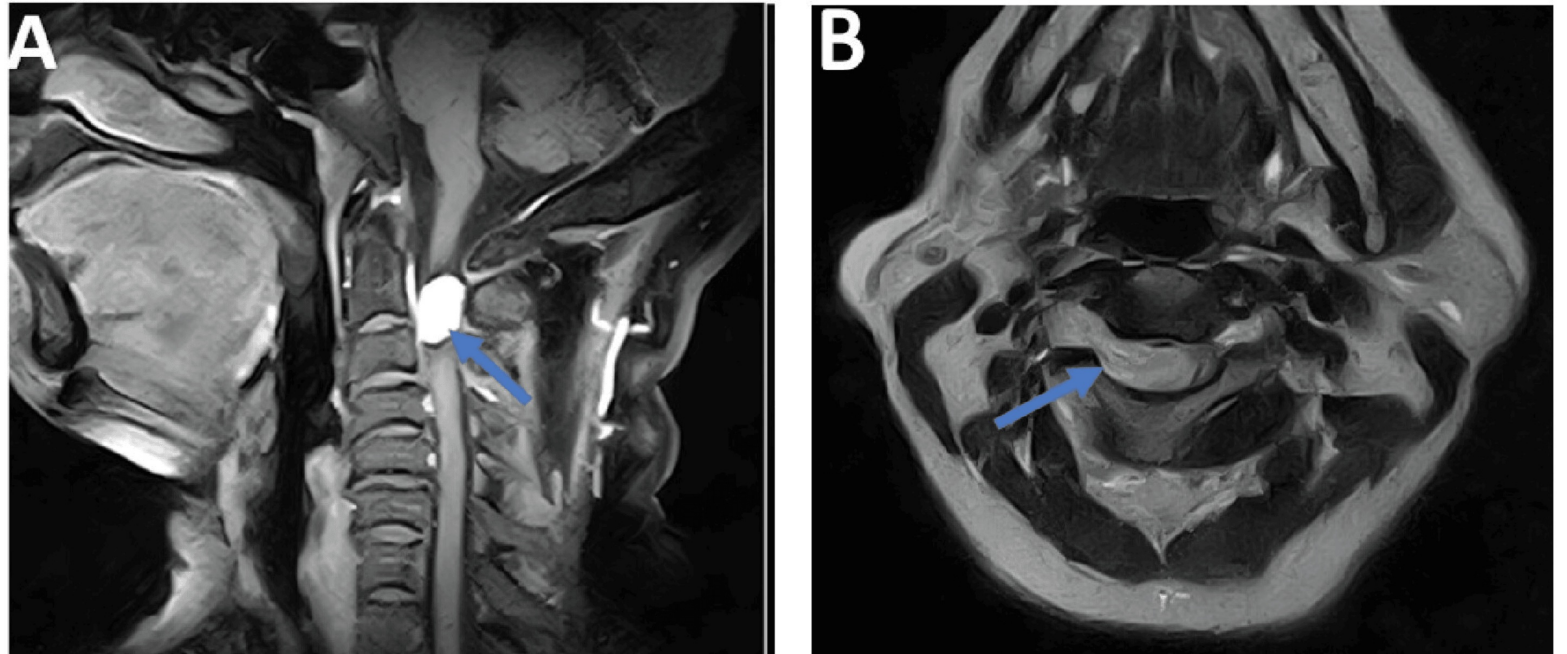
(A) Sagittal T1-weighted MRI image showing a contrast-enhancing intradural mass at the C2–C3 level (blue arrow); (B) Axial T1-weighted MRI image showing a right anterolateral intradural extramedullary mass in contact with the C2 nerve root (blue arrow).

Cervical spine MRI revealed a well-defined, contrast-enhancing intradural extramedullary mass (31x27 mm) located anterolaterally at the C2-C3 level. The lesion had a benign appearance, suggestive of an intradural nerve sheath tumor, with a primary differential diagnosis of meningioma versus schwannoma (Figure 2).

Case 3

A 54-year-old male patient (Patient 3) was admitted to the Neurosurgery Department with complaints of neck pain and tetraparesis persisting for over a month. Neurologically, bilateral pyramidal syndrome was observed. Motor strength was graded at 4/5 in all four limbs. No visible atrophy was noted in the lower extremities.

**Figure 3:**
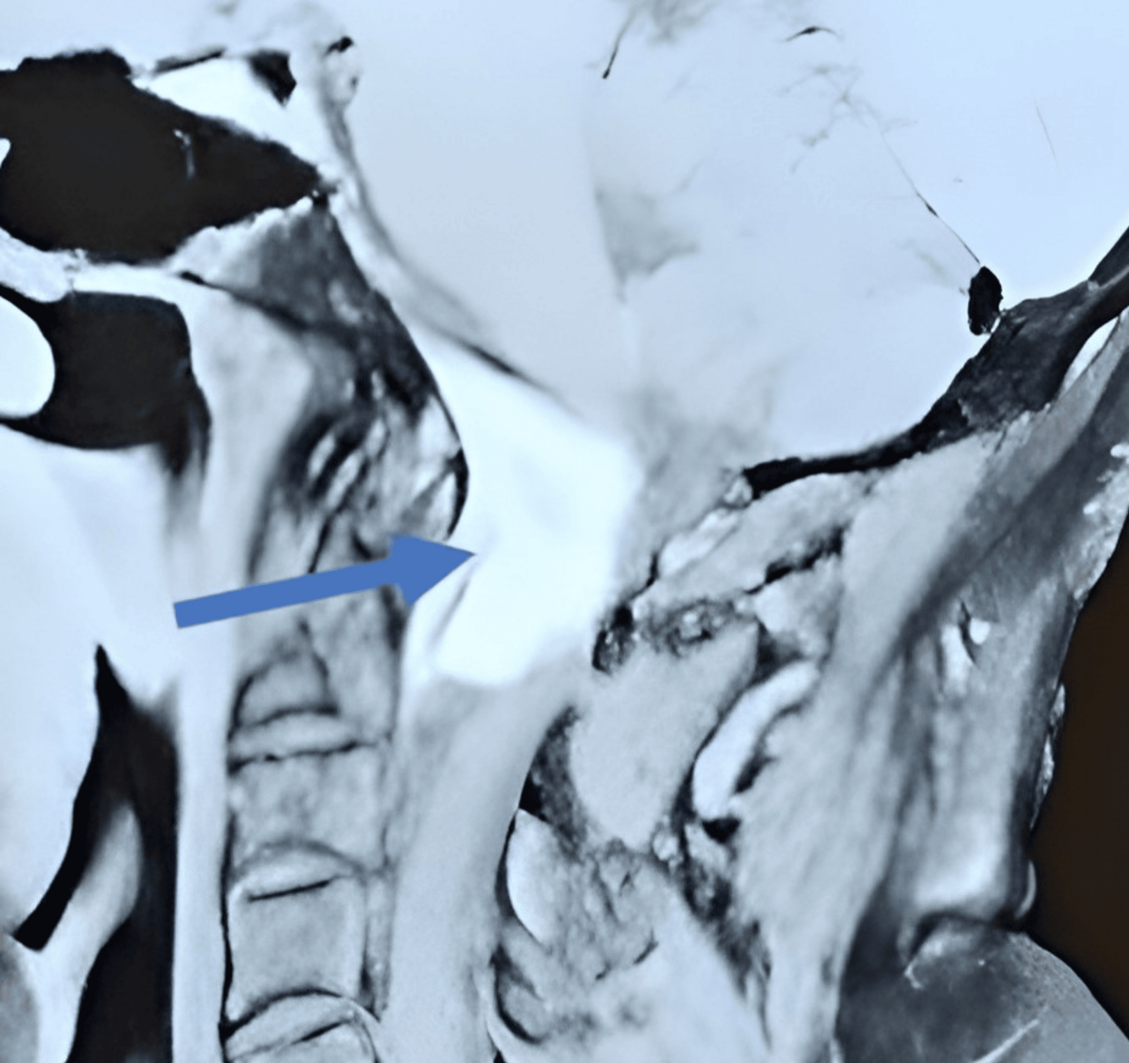
Sagittal T1-weighted MRI showing an extradural mass at the C1–C2 level (blue arrow).

MRI revealed spinal cord compression at the C1-C2 level caused by a mass occupying the extradural space, measuring (35 x 26 mm), with a spontaneous signal on the anterior aspect of the dural sac (Figure 3). A posterior approach for tumor resection was indicated. However, due to financial constraints, the patient was discharged and returned home. Four months later, he was readmitted with worsened symptoms, including cervical pain, paraplegia (muscle strength 2/5), and urinary retention, necessitating hospitalization.

Surgical procedure, intraoperative findings, and postoperative management

**Figure 4:**
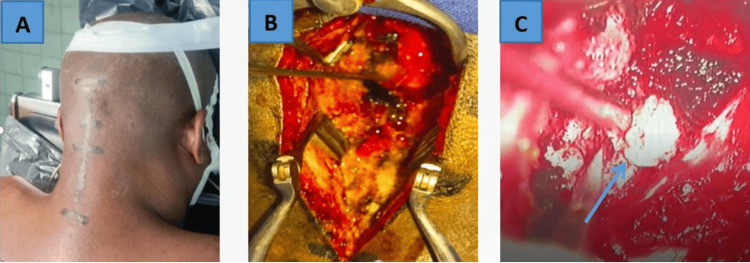
Intraoperative images of the high cervical mass: (A) Marking of the surgical site, (B) Muscular dissection, (C) Tumor excision under neurosurgical microscope (blue arrow).

For all three patients, tumor resection via a posterior approach was indicated and performed. Under general anesthesia, the patient was placed in the prone position, with the chest and pelvis supported by bolsters, and the head secured in a Mayfield headrest (Integra LifeSciences Holdings Corporation, Princeton, New Jersey, United States). The cervicothoracic region was cleansed with soap and running water. The surgical site was marked using an indelible marker (C0 to C5) (Figure 4). Skin preparation was performed with 10% povidone-iodine solution, followed by sterile draping.

For the surgical procedure, we used an orthopedic retractor, a rust retractor, macro-discectomy instruments, hooks, Kerrison rongeurs, gouge clamps, monopolar and bipolar cautery, suction cannulas, and a vacuum system. A midline incision was made extending over C1, C2, and C3 up to the occiput. Muscular dissection was carried out until the posterior arches of C1 and C2 were clearly visualized (Figure 4B). Hemostasis was achieved using a bipolar cautery. A laminectomy of C2 was performed, along with a partial resection of the posterior arch of C1 (approximately 1 cm lateral to the midline).

Intraoperative findings revealed a solid, intradural-extramedullary tumor attached to the right anterior C2 nerve root in the first two patients and to the left anterior C2 nerve root in the third patient. Tumor resection was carried out under the operating microscope (Figure 4C). For microsurgical resection, we used a micro-dissector, micro-curette, and both straight and curved micro-scissors. Hemostasis was secured using Surgicel® (Ethicon, Inc., Raritan, New Jersey, United States). A subfascial suction drain was placed, and closure was performed in anatomical layers, followed by application of a sterile dressing. Intraoperative blood loss was moderate in the first case, with a surgical duration of four hours. In the second and third cases, operative duration was three hours each, with minimal blood loss.

Postoperatively, cervical protection was ensured with a rigid collar worn for four to six weeks, followed by replacement with a semi-rigid collar. The postoperative courses were uneventful in all three cases. All patients recovered normal urination and ambulation, with muscle strength graded 5/5 in both upper and lower limbs, after bladder retraining and physiotherapy sessions. Patient 1 demonstrated functional improvement after nine months of follow-up, Patient 2 after seven months, and Patient 3 after five months. 

**Figure 5:**
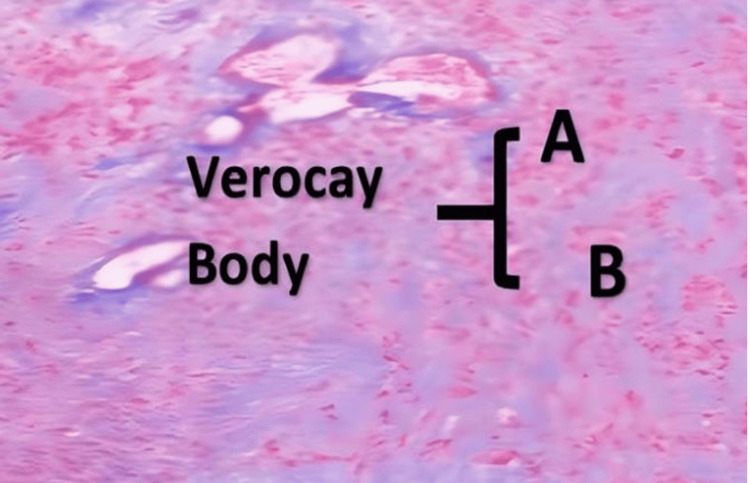
Histopathological image of a schwannoma in Case 1 (hematoxylin and eosin image, 40x objective, showing two cellular areas: Antoni A (a fusocellular proliferation area) and Antoni B (a hypocellular area with myxoid changes).

Histopathological analysis of the resected specimens confirmed the diagnosis of schwannomas (Figure 5).

Table 1 summarizes the three cases.

**Table 1:** Summary of the characteristics and outcomes of the three cases

Patients	Age (years)	Sex	Clinical features	MRI findings	Surgical procedures	Follow-up period	Outcomes
1	46	Male	Neck pain, quadriparesis, constipation, and urinary incontinence. Left Upper limb muscle strength graded: 3/5. Right Upper limb muscle strength graded: 4/5 Lower limbs strength graded: 3/5	Right anterolateral, ovoid tumor (30x28 mm) at C2	Posterior approach, Blood loss moderate, Operative duration: 4 hours	9 months	Improvement of: Neck pain, Neurological function, muscle strength graded: 5/5 for all limbs, Constipation, Urinary Incontinence
2	63	Male	Cervical pain, upper limb weakness, and paresthesia. Left upper limb monoplegia, muscle strength graded: 3/5.	Anterolaterally mass (31x27 mm) at the C2–C3 level	Posterior approach, Blood loss minimal, Operative duration: 3 hours	7 months	Improvement of: Neck pain, Neurological function, muscle strength graded: 5/5 for all limbs
3	54	Female	Beginning: Neck pain, tetraparesis (motor strength: 4/5 all four limbs); After 4 months: Paraplegia (motor strength: 2/5), urinary retention	Mass occupying the extradural space (35 x 26 mm) at C1- C2	Posterior approach, Blood loss minimal, Operative duration: 3 hours	5 months	Improvement of: Neck pain, Neurological function, muscle strength graded: 5/5 for all limbs, Urinary retention

## Discussion

Spinal schwannomas are common tumors, accounting for approximately 30% of primary intradural spinal tumors [14,15]. They primarily arise from Schwann cells, typically originating from the sensory root of spinal nerves [16], and less frequently from the motor root [17]. The frequency and presentation of these tumors vary depending on their spinal location.

Similar to meningiomas, spinal schwannomas are more frequently found in the thoracic spine, where they are typically positioned dorsally or dorsolaterally relative to the spinal cord [18]. The neuromas of the first two cervical nerve roots are uncommon, accounting for only 5.3% of all spinal neuromas and 18% of cervical neuromas [19]. Ventral or ventrolateral positioning of schwannomas, as described in the present cases, is also uncommon, reported in about 5% of cases [20]. C2 nerve root schwannomas (30.9%) are the most commonly involved among the nerve structures in this region. In terms of location relative to the dura, the intradural extramedullary type is most frequent (44.2%), while purely extradural or mixed intra-extradural or intramedullary locations, as in our cases, are rare [2].

According to most authors, spinal schwannomas typically occur between the ages of 30 and 50 years, most commonly in the fourth decade [14]. Our first patient (46 years old) fits within this age range, while the other two were slightly older. Regarding sex distribution, the literature remains inconsistent. Some series report a female predominance [14,15], while others show a higher incidence in males [21]. In our series, two patients were male and one was female.

Spinal schwannomas, like other spinal tumors, often present with a clinical picture of slowly progressive spinal cord compression. The clinical latency depends on several factors, including tumor location, size, growth dynamics, and episodes of tumor expansion or regression [14]. The upper cervical spinal canal is relatively wide, requiring significant tumor growth before clinical symptoms appear [19,22].

Spinal cord compression typically manifests with a combination of spinal, radicular (segmental), and sublesional syndromes. The most indicative symptoms are spinal and radicular pain, reflecting segmental involvement [23]. This segmental syndrome indicates involvement of the specific spinal segment compressed by the tumor, offering valuable localization clues for identifying the level of compression [24]. Involvement of long tracts (sublesional syndrome) may lead to sensorimotor deficits and sphincter disturbances [14].

In the reported cases, all tumors exceeded 2.5 cm in both transverse and vertical dimensions, contributing to significant cord compression. Clinically, this was reflected by neck pain, quadriparesis, and urinary disturbances. Since the tumors originated from anterior roots, sensory function was preserved in all patients. Other, less frequent clinical presentations include normal pressure hydrocephalus, intracranial hypertension, acute spinal cord compression, and spinal subarachnoid hemorrhage [14].

Microsurgical resection remains the gold standard treatment for spinal schwannomas, aiming to relieve compression, preserve anatomical and functional nerve continuity, improve symptoms, and ensure local control [2,14]. Stereotactic radiosurgery may be considered for inoperable patients or for residual or recurrent tumors [25,26]. Endoscopic resection has also recently been explored [24].

Various surgical approaches, anterior, lateral, and posterior, have been described, each with its advantages, limitations, and degrees of exposure. The choice of approach depends on the tumor’s location, level, and relation to dural compartments. In some cases, combined approaches may be necessary for optimal access [27]. Spinal instrumentation may also be required for stabilization [14]. Schwannomas located anteriorly or anterolaterally at the CVJ can often be safely resected via a posterior approach. This route offers sufficient access to both intraspinal and extraspinal components without risking postoperative spinal instability, given the anatomical absence of an intervertebral disc between C1 and C2 and the naturally wide C1-C2 space [19].

The main challenge with the posterior approach is preserving neurovascular structures, particularly the vertebral artery, which courses along the superior margins of the posterior arch of the atlas. It is recommended not to exceed 1 cm of resection on either side of the midline. In our three cases, we used the posterior approach, resecting the posterior arch of the atlas approximately 1 cm on each side of the midline following C2 laminectomy.

Post-surgery clinical outcomes proved that safe surgical resection leads to high rates of symptom improvement in patients with CVJ schwannomas. However, prognosis can be negatively impacted by delayed diagnosis, incomplete tumor resection [28], motor deficits related to nerve root involvement, and postoperative spinal instability [14,29]. In our series, the use of a cervical rigid collar for about one month reduced the risk of postoperative instability to some degree.

In all three cases reported here, the immediate postoperative outcomes were favorable, with full recovery of motor and bladder functions following physiotherapy and bladder rehabilitation, confirming a tendency for favorable outcomes of surgical treatment of giant schwannomas [30].

## Conclusions

This case series highlights the complexity of posterior surgical resection of anterior or anterolateral schwannomas at the CVJ. A better understanding of the anatomy of this region is a major asset for the success of the surgery. This report also shows, although rare, the existence of schwannomas affecting the anterior nerve root. It demonstrates the favorable prognosis associated with complete microsurgical excision of giant schwannomas, even in this anatomically challenging region. External immobilization using a rigid collar, as well as physiotherapy sessions, is essential postoperatively. Posterior approaches, when carefully executed, can provide safe access and yield excellent clinical outcomes.

## References

[1] Pop MM, Bouros D, Klimko A, Pop LA, Topal P, Topal A, Florian IS (2024). Tumor-like lesions in the craniovertebral junction: a case series, systematic review, and meta-analysis. Cancers (Basel).

[2] Palmisciano P, Ferini G, Watanabe G (2022). Surgical management of craniovertebral junction schwannomas: a systematic review. Curr Oncol.

[3] Kerolus MG, O’Toole JE (2020). Special anatomical zone: craniocervical junction tumors. Surgical Spinal Oncology.

[4] Maestro VM, Berthe A (1985 ). Biomechanics of the craniospinal junction [Article in French]. Ann Kinésithér.

[5] Louis DN, Perry A, Wesseling P (2021). The 2021 WHO classification of tumors of the central nervous system: a summary. Neuro Oncol.

[6] Sridhar K, Ramamurthi R, Vasudevan MC, Ramamurthi B (2001). Giant invasive spinal schwannomas: definition and surgical management. J Neurosurg.

[7] Quillo-Olvera J, Lin GX, Kim JS (2018). Severe spinal cord compression by pure giant intradural schwannoma of cervical spine. World Neurosurg.

[8] Jiang H, He J, Zhan X, He M, Zong S, Xiao Z (2015). Occipito-cervical fusion following gross total resection for the treatment of spinal extramedullary tumors in craniocervical junction: a retrospective case series. World J Surg Oncol.

[9] Goel A, Kaswa A, Shah A, Rai S, Gore S, Dharurkar P (2018). Extraspinal-interdural surgical approach for c2 neurinomas-report of an experience with 50 cases. World Neurosurg.

[10] Moini J, Avgeropoulos NG, Samsam M (2021). Schwannoma. Epidemiology of Brain and Spinal Tumors.

[11] Karam YR, Menezes AH, Traynelis VC (2010). Posterolateral approaches to the craniovertebral junction. Neurosurgery.

[12] El Ahmadieh TY, Haider AS, Cohen-Gadol A (2021). The far-lateral suboccipital approach to the lesions of the craniovertebral junction. World Neurosurg.

[13] Wu X, Li D, Zhang L, Li Y (2025). A giant spinal schwannoma at the C1-C2 level: a case report. Medicine (Baltimore).

[14] Himmiche M, Joulali Y, Benabdallah IS, Benzagmout M, Chakour K, Chaoui MF (2019). Spinal schwannomas: case series [Article in French]. Pan Afr Med J.

[15] Seppälä MT, Haltia MJ, Sankila RJ, Jääskeläinen JE, Heiskanen O (1995). Long-term outcome after removal of spinal schwannoma: a clinicopathological study of 187 cases. J Neurosurg.

[16] (2012). Schmidek and Sweet Operative Neurosurgical Techniques: Indications, Methods, and Results. Philadelphia: W. B. Saunders Co.

[17] McLendon R, Rosenblum M, Bigner D (2006). Russell & Rubinstein's Pathology of Tumors of the Nervous System, 7th Ed. J Neurol Neurosurg Psychiatry.

[18] Conti P, Pansini G, Mouchaty H, Capuano C, Conti R (2004). Spinal neurinomas: retrospective analysis and long-term outcome of 179 consecutively operated cases and review of the literature. Surg Neurol.

[19] Kabatas S, Cansever T, Yilmaz C, Demiralay E, Celebi S, Caner H (2010). Giant craniocervical junction schwannoma involving the hypoglossal nerve: case report. Turk Neurosurg.

[20] Yamahata H, Yamaguchi S, Mori M, Kubo F, Tokimura H, Arita K (2013). Ventral schwannoma of the thoracolumbar spine. Asian Spine J.

[21] Argiti K, Fung C, Shah MJ, Vasilikos I, Schnell O, Beck J, Rahal AE (2023). Spinal schwannoma and ependymoma: a diagnosis that shouldn't be missed in SAH - literature review and case report. Neurochirurgie.

[22] Yoon S, Park H, Lee KS, Park SW, Hong CK (2016). Single-stage operation for giant schwannoma at the craniocervical junction with minimal laminectomy: a case report and literature review. Korean J Spine.

[23] Mireau E, Filho GD, Gaudart S (2009). Slow spinal cord compression [Article in French]. EM Consulte.

[24] Feldman M, Kimmell KT, Replogle RE (2015). Resection of an occipital-cervical junction schwannoma through a modified minimally invasive approach: technical note. Surg Neurol Int.

[25] Pollock BE, Foote RL, Stafford SL (2002). Stereotactic radiosurgery: The preferred management for patients with nonvestibular schwannomas?. Int J Radiat Oncol.

[26] Peker S (2019). Non-vestibular schwannoma radiosurgery. Prog Neurol Surg.

[27] Banczerowski P, Lipóth L, Vajda J, Veres R (2003). Surgery of ventral intradural midline cervical spinal pathologies via anterior cervical approach: our experience. Ideggyogy Sz.

[28] Ashour A, Rautenberg M, Buhl R, Mehdorn HM (1999). Giant ventral intradural extramedullary neuroma: case report. Neurosurg.

[29] Yamane K, Takigawa T, Tanaka M, Osaki S, Sugimoto Y, Ozaki T (2013). Factors predicting clinical impairment after surgery for cervical spinal schwannoma. Acta Med Okayama.

[30] Handa K, Ozawa H, Aizawa T, Hashimoto K, Kanno H, Tateda S, Itoi E (2019). Surgical management of giant sacral schwannoma: a case series and literature review. World Neurosurg.

